# Engineered fibroblast growth factor 19 protects from acetaminophen-induced liver injury and stimulates aged liver regeneration in mice

**DOI:** 10.1038/cddis.2017.480

**Published:** 2017-10-05

**Authors:** Gloria Alvarez-Sola, Iker Uriarte, Maria U Latasa, Maddalen Jimenez, Marina Barcena-Varela, Eva Santamaría, Raquel Urtasun, Carlos Rodriguez-Ortigosa, Jesús Prieto, Fernando J Corrales, Anna Baulies, Carmen García-Ruiz, Jose C Fernandez-Checa, Pedro Berraondo, Maite G Fernandez-Barrena, Carmen Berasain, Matías A Avila

**Affiliations:** 1 CIBERehd, Instituto de Salud Carlos III, Clinica Universidad de Navarra, Avda, Pio XII, n 36, Pamplona 31008, Spain; 2 Hepatology Programme, CIMA, Idisna, Universidad de Navarra, Avda, Pio XII, n 55, Pamplona 31008, Spain; 3 CIBERehd, Instituto de Salud Carlos III, Barcelona, Spain; 4 Department of Cell Death and Proliferation, Instituto de Investigaciones Biomédicas de Barcelona, CSIC and Liver Unit-Hospital Clinic-IDIBAPS, Barcelona, Spain; 5 Research Center for ALPD, Keck School of Medicine, University of Southern California, Los Angeles 90033, CA, USA; 6 Immunology and Immunotherapy Programme, CIMA, Idisna, Universidad de Navarra, Avda, Pio XII, n 55, Pamplona 31008, Spain

## Abstract

The liver displays a remarkable regenerative capacity triggered upon tissue injury or resection. However, liver regeneration can be overwhelmed by excessive parenchymal destruction or diminished by pre-existing conditions hampering repair. Fibroblast growth factor 19 (FGF19, rodent FGF15) is an enterokine that regulates liver bile acid and lipid metabolism, and stimulates hepatocellular protein synthesis and proliferation. FGF19/15 is also important for liver regeneration after partial hepatectomy (PH). Therefore recombinant FGF19 would be an ideal molecule to stimulate liver regeneration, but its applicability may be curtailed by its short half-life. We developed a chimaeric molecule termed Fibapo in which FGF19 is covalently coupled to apolipoprotein A-I. Fibapo retains FGF19 biological activities but has significantly increased half-life and hepatotropism. Here we evaluated the pro-regenerative activity of Fibapo in two clinically relevant models where liver regeneration may be impaired: acetaminophen (APAP) poisoning, and PH in aged mice. The only approved therapy for APAP intoxication is *N*-acetylcysteine (NAC) and no drugs are available to stimulate liver regeneration. We demonstrate that Fibapo reduced liver injury and boosted regeneration in APAP-intoxicated mice. Fibapo improved survival of APAP-poisoned mice when given at later time points, when NAC is ineffective. Mechanistically, Fibapo accelerated recovery of hepatic glutathione levels, potentiated cell growth-related pathways and increased functional liver mass. When Fibapo was administered to old mice prior to PH, liver regeneration was markedly increased. The exacerbated injury developing in these mice upon PH was attenuated, and the hepatic biosynthetic capacity was enhanced. Fibapo reversed metabolic and molecular alterations that impede regeneration in aged livers. It reduced liver steatosis and downregulated *p21* and hepatocyte nuclear factor 4 *α* (*Hnf4α*) levels, whereas it stimulated *Foxm1b* gene expression. Together our findings indicate that FGF19 variants retaining the metabolic and growth-promoting effects of this enterokine may be valuable for the stimulation of liver regeneration.

The liver fulfils an essential role in the detoxification of metabolites and exogenous compounds. In this process, noxious stimuli may be generated, eventually causing hepatocellular death. To cope with this situation the liver has developed an extraordinary regenerative capacity unparalleled by other organs.^1^ Liver regeneration also manifests as a vigorous response after surgical removal of part of the tissue.^2, 3^ Therefore, in the context of parenchymal damage or tissue resection a potent hepatoprotective and regenerative reaction is triggered within the liver as well as systemically.^1, 4^ Liver regeneration involves survival and pro-mitogenic mechanisms activated by fluctuating metabolites, cytokines and growth factors in an intricate cellular and molecular crosstalk.^1, 2^ The regenerative response usually concludes with the restoration of histological integrity and hepatic function, which is essential for homeostasis and survival.^5, 6^ However, there are circumstances in which the regenerative capacity of the liver is overwhelmed by the extent of parenchymal destruction, or diminished by pre-existing conditions that amplify injury and hamper tissue repair.^7^

In the clinical context, acute liver injury leading to impairment of liver function and regenerative capacity may occur under different situations. Among them drug intoxication is a leading cause, with acetaminophen (APAP) overdose accounting for almost 50% of all cases in the western world.^8^ Currently, the only approved drug to treat APAP intoxication is *N*-acetylcysteine (NAC).^8, 9^ The efficacy of NAC is mostly based on its positive effects on hepatic glutathione (GSH) levels, which are depleted by the toxic APAP metabolite *N*-acetyl-*p*-benzoquinone (NAPQI). NAPQI triggers mitochondrial dysfunction, oxidative stress and massive hepatocyte necrosis.^10^ Hepatocellular death unlashes the activation of the innate immune system, a reaction increasingly recognized as protective and pro-regenerative rather than a deleterious response.^9^ Clinical observations indicate that regeneration of new parenchymal cells appears essential for survival after APAP-induced acute liver failure.^11^ Therefore, understanding the endogenous mechanisms of liver protection and regeneration may allow the identification of therapeutic strategies.^12, 13^ In this line of thinking, enhancement of the natural regenerative response by the experimental administration of growth factors and cytokines has recently shown promising effects on APAP toxicity.^14, 15, 16, 17, 18^ Exploring new therapeutic approaches is important, as NAC is only effective when administered early after APAP intoxication and liver transplantation is the sole therapeutic alternative in severe cases.^10, 13^

Another clinical scenario in which liver regeneration may fail is when partial hepatectomy (PH) is performed in diseased or aged livers for the removal of primary or metastasic tumors, or after segmental liver transplantation.^7^ For instance, patients with fatty livers, a condition usually accompanied by cholestasis, frequently present a worse outcome after resection.^19, 20^ This impaired response has been widely validated in experimental models of hepatosteatosis.^21, 22^ Similarly, liver regeneration is compromised in elderly patients and aged rodents.^23, 24, 25^ The mechanisms underlying the decline in the regenerative capacity of aged livers are not completely known, although some epigenetic and intracellular signaling mechanisms leading to cell cycle alterations have been exposed.^23, 26, 27, 28, 29, 30^ Unraveling these fundamental processes may lead to the development of pharmacological therapies to alleviate age-related liver regeneration defects, as experimentally demonstrated in recent studies.^23, 29, 31^ With an increasingly aging population it is important to devise such therapies.

We and others have recently shown the significant role played by the enterokine fibroblast growth factor 19 (FGF19; FGF15 in rodents)^32, 33^ and its receptor FGFR4 in liver regeneration after PH.^34, 35, 36^ FGF15/19 controls bile acids (BA) homeostasis, reduces liver fat accumulation and enhances hepatocyte survival and proliferation. FGF15/19 delivery from viral vectors improves mouse survival after extensive liver resection,^22, 34^ and recombinant FGF19 protects from cholestatic liver injury and lipotoxicity.^22, 37, 38^ From a translational perspective these characteristics identify FGF15/19 as a promising tool to improve liver regeneration under unfavorable conditions. However, FGF15/19 has a very short half-life with a high glomerular filtration rate.^38^ To circunvent this limitation we recently developed a chimaeric molecule based on the fusion of FGF19 with apolipoprotein A-I (ApoA-I) named Fibapo.^22^ The ApoA-I moiety confers Fibapo increased biological stability, and targets it to the liver through the interaction of the ApoA-I moiety with the hepatocyte's scavenger receptor class B type I.^22^ Here, we demonstrate the applicability of Fibapo for the treatment of APAP-induced liver injury and in the stimulation of aged liver regeneration.

## Results

### Fibapo protects from APAP toxicity and enhances liver regeneration

**Figure 1:**
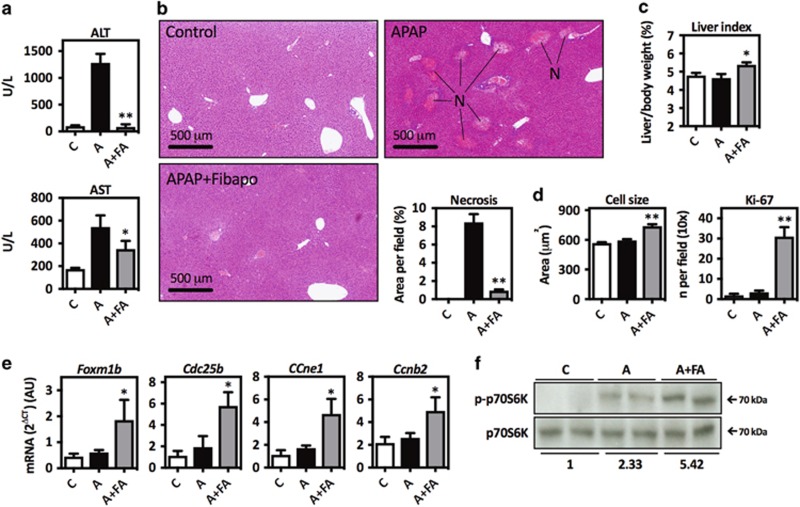
Fibapo administration protects from APAP-induced liver injury. (**a**) Serum levels of ALT and AST in control mice (C), mice that were treated with APAP (A) (300 mg/kg) or mice that 2, 10 and 24 h after APAP injection received three doses of Fibapo (A+FA) (*n*=7 mice per group). Blood and tissue samples were taken 36 h after APAP administration, when mice were killed. (**b**) Representative H&E-stained liver tissue sections from mice treated as described. Necrotic areas are indicated (N). Graph shows the quantification of tissue necrotic areas. (**c**) Liver weight to body weight ratio (liver index) in mice from the different treatment groups described. (**d**) Left panel shows the average hepatocyte size and right panel shows the quantification of hepatocytes with Ki-67-positive nuclei in liver tissue sections from mice treated as described. (**e**) Quantitative PCR analysis of the expression of cell cycle-related genes in liver tissues from mice treated as described. (**f**) Western blot analysis of phospho-p-70S6K (p-p-70S6K) and total p70S6K levels in liver tissue samples from mice treated as described. Representative blots are shown. *P<0.05 and **P<0.01 *versus* APAP-treated mice. AU: arbitrary units

To analyze the effect of Fibapo on APAP toxicity, mice received three doses of the recombinant protein 2, 10 and 24 h after APAP (300 mg/kg) injection. Serum transaminases levels and centrilobular liver necrosis were reduced by Fibapo administration (Figures 1a and b). Liver injury elicited by APAP is followed by a compensatory regenerative response.^39^ We found a clear increase in liver weight to body weight ratio (Figure 1c), hepatocyte cell size and hepatocellular proliferation (Ki-67 labeling) upon Fibapo treatment (Figure 1d and Supplementary Figure 1). Consistently, the expression of cell cycle regulatory genes *Foxm1b*, *Cdc25b*, *Ccne1* and *Ccnb2* was induced (Figure 1e and Supplementary Figure 2). To a great extent, FGF19 regulates hepatocellular proliferation and growth through the activation of the mechanistic target of rapamycin complex 1 (mTORC1)-p-70S6 kinase (p70S6K) pathway.^22, 40^ We observed that mice treated with Fibapo after APAP administration showed increased p-p70S6K levels (Figure 1f). The hepatic expression levels of interleukin 10, a cytokine involved in the endogenous hepatoprotective response to APAP intoxication,^41^ was also markedly induced (Supplementary Figure 3).

**Figure 2:**
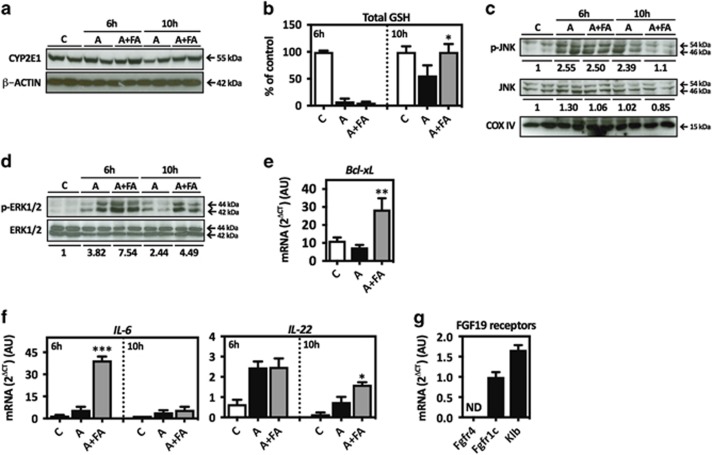
Effects of Fibapo on early hepatic responses to lethal APAP doses. (**a**) Western blot analysis of CYP2E1 expression in the liver of mice (*n*=7 per group) treated with APAP (A) (500 mg/kg) that 2 h later received a single dose of Fibapo (FA). Mice were killed 6 and 10 h after APAP injection. Representative blots are shown. (**b**) Total glutathione (GSH) levels in the livers of mice treated as described and killed at 6 h and 10 h after APAP injection. (**c**) Western blot analysis of phospho-JNK (p-JNK) and total JNK levels in mitochondrial fractions obtained from livers of mice treated as described and killed at 6 and 10 h after APAP injection. COX IV protein levels were analyzed as loading control. Representative blots are shown. (**d**) Western blot analysis of phospho-ERK1/2 (p-ERK1/2) and total ERK1/2 levels in liver homogenates obtained from mice treated as described and killed at 6 and 10 h after APAP administration. Representative blots are shown. (**e**) Quantitative PCR analysis of *Bcl-xL* mRNA levels in the liver of mice treated as described and killed at 10 h after APAP administration. (**f**) Quantitative PCR analysis of *IL-6* and *IL-22* mRNA levels in the liver of mice treated as described and killed at 6 h and 10 h after APAP administration. (**g**) Quantitative PCR analysis of the mRNA levels of the FGF19 receptors FGFR4, FGFR1c and co-receptor Klb in primary cultured murine macrophages. AU: arbitrary units. ND: not detected. *P<0.05, **P<0.01 and ***P<0.001 *versus* APAP (A)-treated mice

To further test the hepatoprotective effects of Fibapo we treated mice with a higher dose of APAP (500 mg/kg), which induces significant mortality (see below). Two hours after APAP injection mice received a single dose of Fibapo and were killed 6 and 10 h later. In APAP hepatotoxicity, the critical initial step involves GSH depletion owing to conjugation with NAPQI, the reactive metabolite of APAP.^10^ The principal enzyme in NAPQI formation is cytochrome 2E1 (*Cyp2E1*).^9^ CYP2E1 expression was unchanged by Fibapo (Figure 2a). Although the early depletion of hepatic GSH was not prevented by Fibapo, GSH contents were restored to control levels by 10 h (Figure 2b). These observations suggest that Fibapo would not affect APAP metabolism nor the initial phases of APAP toxicity,^10^ but may help the functional recovery of hepatocytes as indicated by GSH levels.

Sustained activation and mitochondrial translocation of c-jun-*N*-terminal protein kinase (JNK), leading to loss of mitochondrial potential and ATP production, has been mechanistically related to APAP hepatotoxicity.^9, 42^ Consistently, we observed that APAP increased JNK phosphorylation and mitochondrial translocation (Figure 2c). Although at 6 h post APAP administration p-JNK levels were not affected by Fibapo these were significantly reduced by 10 h (Figure 2c). Concomitantly, increased activation of extracellular signal-regulated kinase 1/2 (ERK1/2), considered part of the compensatory pro-regenerative signaling triggered by APAP toxicity,^39, 43^ was observed in Fibapo-treated mice (Figure 2d). Expression of *Bcl-xL*, an inhibitor of mitochondrial-dependent cell death involved in hepatoprotection from APAP-induced injury,^14, 42^ was enhanced upon Fibapo treatment (Figure 2e).

IL-6 upregulation is detected in APAP-intoxicated mice, and is considered part of the endogenous protective response.^41^ In mice treated with Fibapo hepatic IL-6 expression was markedly potentiated (Figure 2f). Expression of the IL-6-related hepatoprotective cytokine IL-22^41^ was also sustained upon Fibapo treatment (Figure 2f). Induction of IL-6 by FGF19 administration has been recently reported in *db/db* mice.^44^ The cellular source of IL-6 in these animals were liver infiltrating myeloid cells, however the precise mechanism by which FGF19 elicited IL-6 production was not evaluated.^44^ FGF19 signals through FGFR4 and FGFR1 isoform c (FGFR1c), and the coexpression of the membrane protein *β*-Klotho (Klb) is absolutely required for effective signaling.^45^ We examined the expression of these receptors in primary cultured mouse macrophages. Interestingly, we found that these cells expressed FGFR1c and Klb, but not FGFR4 (Figure 2g).

### Late administration of Fibapo protects from lethal doses of APAP and stimulates parenchymal regeneration

**Figure 3:**
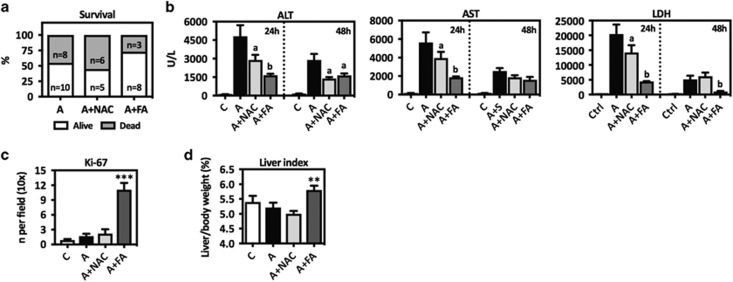
Late administration of Fibapo protects from lethal doses of APAP and stimulates liver regeneration. (**a**) Mice were treated with APAP (A) (500 mg/kg) and 6 and 24 h later received two doses of either NAC (A+NAC) or Fibapo (A+FA). Graph shows total mouse survival at 48 h after APAP intoxication. (**b**) Serum levels of liver enzymes in mice treated as described and measured at 24 and 48 h after APAP intoxication. (**c**) Quantification of hepatocytes with Ki-67-positive nuclei as determined by immunohistochemical analysis performed in liver tissue sections from mice treated as described. Liver tissue samples were obtained from surviving mice 48 h after APAP administration. (**d**) Liver weight to body weight ratio (liver index) in mice from the different treatment groups described. This parameter was measured in mice surviving 48 h after APAP administration. ^a^*P*<0.05 *versus* A, ^b^*P*<0.05 *versus* A+NAC, **P<0.01 *versus* A+NAC, ***P<0.001 *versus* A+NAC

To better mimic the clinical situation, in which patients are treated at late times after APAP poisoning, mice received Fibapo or NAC at 6 and 24 h after APAP. In this case we used 500 mg/kg of APAP, a dose that causes significant mortality and may compromise the endogenous regenerative response.^39^ Under these conditions NAC treatment did not improve mouse survival, whereas mice receiving Fibapo showed less mortality and lower levels of circulating liver enzymes (Figures 3a and b). Increased hepatocellular proliferation (Ki-67 labeling) (Figure 3c and Supplementary Figure 4) and liver weight to body weight ratio (Figure 3d) were also observed.

### Fibapo improves liver regeneration in aged mice

**Figure 4:**
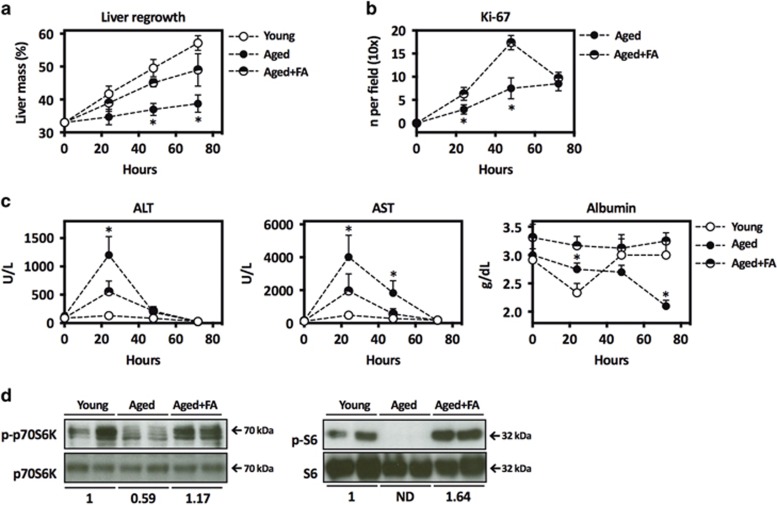
Fibapo improves liver regeneration in aged mice. (**a**) Recovery of liver mass after 66% PH in young mice, aged mice and aged mice that received three injections of Fibapo (FA) on 3 consecutive days before surgery (*n*=6 mice per group). (**b**) Quantification of hepatocytes with Ki-67-positive nuclei as determined by immunohistochemical analysis performed in liver tissue sections from aged mice and aged mice treated with Fibapo (FA) at the indicated time points after PH. (**c**) Circulating levels of liver enzymes and albumin in serum from young mice, aged mice and aged mice treated with Fibapo (FA) measured at the indicated time points after PH. (**d**) Western blot analysis of phospho-S6 (p-S6) levels (left panel) and p-p70S6K levels (right panel) in the livers of young mice, aged mice and aged mice treated with Fibapo (FA). Liver samples were analyzed 3 h after Fibapo administration. Representative blots are shown. ND: not detected. *P<0.05 *versus* aged mice treated with Fibapo

We recently reported that Fibapo significantly ameliorates the regeneration of steatotic livers.^22^ Aged mice also show impaired regeneration, so we tested the effect of Fibapo in old mice undergoing PH. Fibapo was administered for 3 consecutive days before resection. As described we observed that liver regrowth post PH was markedly reduced in old mice,^46^ whereas this response was significantly enhanced by Fibapo (Figures 4a and b). Parenchymal injury, which is exacerbated in aged rodents after PH,^46, 47^ was likewise reduced by Fibapo (Figure 4c). Interestingly, the hepatic biosynthetic capacity was also markedly enhanced, as indicated by serum albumin concentrations (Figure 4c). Consistently, upon Fibapo injection we detected increased p-p70S6K levels and a robust activation of S6 phosphorylation, which link FGF19 signaling to protein synthesis in liver cells (Figure 4d).^32, 40^

### Fibapo treatment corrects biochemical and molecular defects associated with the impairment of liver regeneration in aged mice

**Figure 5:**
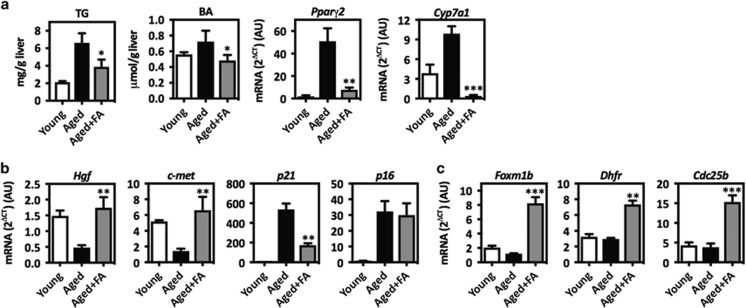
Fibapo improves metabolic parameters and molecular alterations associated with the impairment of liver regeneration in aged mice. (**a**) Analysis of the intrahepatic levels of triglycerides (TG) and bile acids (BA) in young mice, control aged mice and aged mice that received three injections of Fibapo (FA) on 3 consecutive days before surgery (*n*=6 mice per group). Quantitative PCR analysis of the levels of *Pparg2* and *Cyp7a1* mRNAs in the liver of young, aged and aged mice treated as described. (**b**) Quantitative PCR analysis of the levels of *Hgf*, *c-met, p21* and *p16* mRNAs in the liver of young, aged and aged mice treated as described. (**c**) Quantitative PCR analysis of the levels of *Foxm1b*, *Dhfr* and *Cdc25b* mRNAs in the liver of young, aged and aged mice treated as described. *P<0.05, **P<0.01 and ***P<0.001 *versus* control aged mice

Steatosis, commonly found in aged livers,^47, 48^ and the presence of steatosis-associated cholestasis^22, 49^ are related to impaired regeneration after PH.^19, 20^ Accordingly, aged mice had increased levels of intrahepatic triglycerides (TG) and total BA compared with young mice (Figure 5a). In agreement with the potent regulatory effects of Fibapo on hepatic lipid and BA metabolism,^22, 33^ intrahepatic TG and BA levels were significantly reduced (Figure 5a). Mechanistically, Fibapo inhibited the expression of peroxisome proliferator-activated receptor *γ* variant 2 and cytochrome 7a1 genes, key mediators of hepatic TG accumulation and BA synthesis, respectively^50, 51^ (Figure 5a).

Impaired aged liver regeneration has been linked to defective expression of genes involved in growth factor signaling and cell cycle regulation.^23, 30^ We observed that the reduced expression of hepatocyte growth factor (*Hgf*) and its receptor *c-met* found in the livers of aged mice^25, 46^ was reversed by Fibapo (Figure 5b). As described, we also detected increased mRNA levels of the cell cycle inhibitors p21 and p16 in aged livers.^46, 52^ *p21* overexpression was significantly corrected by Fibapo, however *p16* remained unaltered (Figure 5b and Supplementary Figure 5). Among the best-characterized mechanisms involved in the impairment of aged liver regeneration is the transcriptional repression of E2F-dependent genes.^23^ This was shown for key cell proliferation-related genes like *Foxm1b* and *Dhfr*.^53, 54^ *Foxm1b* and *Dhfr* expression was markedly increased in aged mice treated with Fibapo (Figure 5c). Consistent with the strong induction of *Foxm1b* expression, *Cdc25b*, a Cdk phosphatase important for hepatocyte cell cycle progression and a transcriptional target of Foxm1b,^55^ was also induced (Figure 5c). Accordingly, the expression of proliferating cell nuclear antigen (PCNA) was also increased (Supplementary Figure 5).

### Fibapo regulates hepatocyte nuclear factor 4*α* (HNF4*α*) gene expression

**Figure 6:**
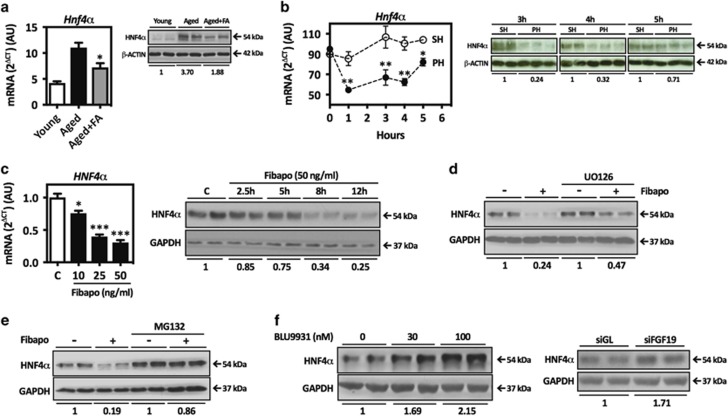
Effect of Fibapo and FGF19/FGFR4 signaling on HNF4*α* gene expression. (**a**) *Hnf4α* mRNA and protein levels in the livers of young, aged and aged mice that received three injections of Fibapo (FA) on 3 consecutive days (*n*=6 mice per group). *Hnf4α* mRNA levels were analyzed by quantitative PCR and *Hnf4α* protein levels by western blotting. Representative blots are shown. (**b**) Quantitative PCR analysis of *Hnf4α* mRNA and western blot analysis of *Hnf4α* protein levels in the liver of young mice at early time points after PH (*n*=5 per time point and condition). Liver tissue samples from mice that underwent laparotomy but were not hepatectomized (sham operated mice, SH) are used as controls. Representative blots are shown. (**c**) Left panel shows *HNF4α* mRNA levels analyzed by quantitative PCR in Hep3B cells treated for 12 h with the indicated concentrations of Fibapo. Right panel shows HNF4*α* protein levels determined by western blotting in Hep3B cells treated with 50 ng/ml of Fibapo for the indicated periods of time. Representative blots are shown. (**d**) Effect of Fibapo on HNF4*α* protein levels in Hep3B cells in the presence of the MEK-ERK signaling inhibitor UO126 analyzed by western blotting. Cells were pre-treated with UO126 (10 *μ*M) for 45 min prior to Fibapo (50 ng/ml) addition and were lysed after 8 h of treatment. Representative blots are shown. (**e**) Effect of Fibapo on HNF4*α* protein levels in Hep3B cells in the presence of the proteasome inhibitor MG-132. Where indicated cells were pre-treated with MG-132 (10 *μ*M) for 45 min and then stimulated with Fibapo for 8 h. Representative blots are shown. (**f**) Left panel shows the effect of basal FGFR4 signaling on HNF4*α* protein levels in Hep3B cells. Cells were treated with the indicated concentrations of the FGFR4-specific inhibitor BLU9931 for 8 h and HNF4*α* protein levels were determined. Right panel shows HNF4*α* protein levels in Hep3B cells transfected with an FGF19-specific siRNA (siFGF19) or a control siRNA (siGL) as determined by western blotting 72 h after transfections. Representative western blots are shown

HNF4*α* is a key transcription factor for the preservation of hepatocyte differentiation and quiescence.^5^ HNF4*α* knockdown in mouse liver results in a strong hepatoproliferative response.^56, 57, 58, 59^ Remarkably, it has been recently reported that hepatic expression of *Hnf4α* is increased in aged rats.^60^ We validated this observation in aged mice, and we also found that Fibapo reduced HNF4*α* mRNA and protein levels (Figure 6a). HNF4*α* transcriptional activity is repressed in the first hours after PH, allowing the induction of early genes mediating the onset of liver regeneration.^61, 62^ Consequently, we evaluated HNF4*α* expression shortly after PH and found a transient but significant downregulation of *Hnf4α* expression during the normal regenerative response in young mice (Figure 6b). These observations suggest that *Hnf4α* downregulation may also contribute to the pro-regenerative activity of Fibapo in aged mice livers. The mechanisms regulating the expression of HNF4*α* in the adult liver are likely to be complex and are not completely known.^5^ Previously, we reported that the splice regulator SLU7 is a fundamental factor in the preservation of hepatocellular differentiation and quiescence, and a positive effector of HNF4*α* gene expression in mouse liver.^63^ Therefore, we determined SLU7 expression in the livers of young and aged mice, as well as in aged mice treated with Fibapo. As observed for HNF4*α* we found that SLU7 mRNA levels were higher in the liver of aged mice and were significantly reduced upon Fibapo administration (Supplementary Figure 6a).

Next, we evaluated whether Fibapo could directly regulate HNF4*α* expression in liver cells. HNF4*α* mRNA and protein levels were reduced in Hep3B cells upon Fibapo treatment (Figure 6c), and this was partially dependent on mitogen-activated protein kinase kinase (MEK)-ERK signaling (Figure 6d and Supplementary Figure 6b). Consistently, FGF19 also reduced HNF4*α* mRNA and protein levels in Hep3B cells (Supplementary Figure 6c). Interestingly, Fibapo-mediated HNF4*α* downregulation was to a great extent proteasome dependent (Figure 6e). The specificity of these findings, and the significance of the FGF19/FGFR4 signaling system in the regulation of HNF4*α* expression, were further supported by the observation that both inhibition of FGFR4 with BLU9931, an FGFR4-specific small molecule inhibitor,^64^ or upon FGF19 knockdown by siRNA transfection, increased basal HNF4*α* protein levels (Figure 6f and Supplementary Figure 6d).

## Discussion

We evaluated the therapeutic potential of Fibapo, an engineered version of FGF19 with improved pharmacokinetics^22^ in two clinically relevant models of acute liver failure and impaired regeneration. In APAP-induced liver injury, Fibapo reduced parenchymal necrosis and promoted a robust regenerative response. Physiologically, FGF19 expression is induced in ileal enterocytes by BA during their enterohepatic circulation, and FGF15 was recently identified as an important endogenous mediator of mouse liver regeneration and parenchymal protection after PH.^34, 35, 36^ The role of endogenous FGF15/19 in APAP toxicity is unknown. However, inhibition of ileal FGF15 expression by removing BA from the enterohepatic circulation, or its upregulation by feeding mice a cholate-supplemented diet, were, respectively, associated with increased or reduced APAP-mediated liver injury.^65^ These findings, together with our current observations of the beneficial effects of Fibapo suggest a cytoprotective and regenerative role for FGF15 in APAP hepatotoxicity. The mechanisms mediating Fibapo hepatoprotection are likely to be diverse. Although it did not inhibit the early depletion of hepatic GSH, recovery of GSH levels was faster in treated mice. Interestingly, a similar effect on GSH levels was observed in APAP-intoxicated mice treated with vascular endothelial growth factor.^15^ In the case of Fibapo, this may reflect an improvement of the overall hepatic metabolic capacities, which would be in agreement with the strong positive effects of FGF19 on nutrient metabolism, protein synthesis and hepatocellular energetics.^32, 40^ In support of this notion we observed increased p70S6K phosphorylation in Fibapo-treated mice. Activation of the mTORC1-p70S6K pathway by FGF19 in the liver has been recently reported.^22, 40^ This pathway mediates robust anabolic effects, including the stimulation of cell growth and proliferation.^66^ Besides p70S6K, we also detected enhanced activation of ERK1/2, a kinase strongly linked to hepatocyte proliferation.^32, 67^ Interestingly Fibapo increased the expression of *Foxm1b*, a transcription factor essential for hepatocyte proliferation but also associated with hepatocellular hypertrophy.^55, 68^ Accordingly, we observed a hyperproliferative and a hypertrophic response in mice that received Fibapo after APAP intoxication. These combined effects may be mechanistically relevant, as liver growth and regeneration are emerging as key determinants of the outcome of APAP intoxication. Consistently, it has been recently shown that inhibition of epidermal growth factor receptor, a key mediator of liver regeneration after PH,^69^ results in impaired regeneration, exacerbated liver injury and high mortality in APAP intoxication.^70^

The pro-regenerative effects of Fibapo were accompanied by reduced parenchymal injury. Prolongued translocation of active JNK to mitochondria is a central event in APAP-induced hepatocellular death.^9, 42^ We observed reduced activation of mitochondrial JNK upon Fibapo administration. The mechanisms involved in this response are currently unknown. JNK activation during APAP-mediated liver injury is strongly related to mitochondrial stress and production of reactive oxygen species.^10, 42^ We could speculate that enhanced recovery of GSH levels by Fibapo could reduce oxidative stress, attenuate JNK activation and therefore limit its mitochondrial translocation.^42^ Nevertheless, further studies are required to delineate the precise mechanisms of this response. Upregulation of Bcl-xL may also contribute to the protective effects of Fibapo, as Bcl-xL has been linked to the hepatoprotective activity of stem cell factor and c-kit in APAP-induced liver injury.^14^

The activation of the innate immune system upon APAP intoxication is increasingly regarded as part of an endogenous protective reaction.^9^ In particular, IL-6 expression appears necessary for liver regeneration.^71^ Intriguingly, we observed that Fibapo administration to APAP-treated mice markedly enhanced IL-6 expression. This suggests that Fibapo could also promote liver regeneration through non-cell autonomous pathways. Our findings are consistent with a recent report showing hepatic IL-6 upregulation upon FGF19 administration to *db/db* mice.^44^ In that study the source of FGF19-triggered IL-6 were liver infiltrating myeloid cells, although the exact mechanism of FGF19-mediated IL-6 induction was not examined.^44^ In view of these and our current observations, we evaluated the expression of the FGF19 receptors FGFR4, FGFR1c and the obligate co-receptor Klb^45^ in murine macrophages. We found that mouse macrophages express FGFR1c and Klb, but not FGFR4 mRNA. To our knowledge, this is the first description of the expression of these receptors in murine innate immune cells, and it suggests that the cellular targets of FGF19, and Fibapo, might be broader than initially thought.

We also demonstrated the therapeutic potential of Fibapo in the context of aged liver regeneration. Interestingly, basal circulating FGF19 levels are lower in older individuals compared with young healthy people.^72^ Given the important regulatory role of FGF19 in liver fat metabolism,^22, 33^ its reduced availability in older persons may underlie the steatosis commonly found in this population.^47, 48^ Steatosis represents a significant obstacle to a successful liver regeneration.^19, 20^ Its attenuation by Fibapo may explain in part the decreased injury and improved liver regeneration found in aged mice. Nevertheless, we believe that this molecule has also more direct actions on genes involved in hepatocellular proliferation. Restoration of *Hgf* and *c-met* expression levels, which are depressed in aged livers,^25, 46^ may be an important effect of Fibapo given the crucial role of the HGF/c-Met system in liver regeneration.^69^

One important molecular barrier in the regeneration of aged livers is the formation of repressive complexes on E2F-dependent promoters, such as those of *Foxm1b* and *Dhfr* genes, which are essential for cell proliferation.^23^ We observed that Fibapo administration induced a robust upregulation of *Foxm1B* and *Dhfr*. Stimulation of *Foxm1b* expression by Fibapo may be a key mechanism of action. Certainly, forced hepatic expression of *Foxm1b*, either from a transgene or an adenoviral vector, improves liver regeneration in old mice,^30, 73^ and the beneficial effects of growth hormone and farnesoid X receptor agonists on aged mouse liver regeneration have been related to *Foxm1b* upregulation.^26, 31, 53^ Moreover, the inhibition of *p21* and the activation of *Cdc25b* gene expression elicited by Fibapo may be also dependent on *Foxm1b* stimulation.^55^ Concomitant with the upregulation of *Foxm1b* we found that Fibapo also reduced the levels of HNF4*α*, a key factor in the preservation of hepatocellular differentiation and quiescence.^5, 56, 58^ The increased expression of HNF4*α* recently found in aged livers,^60^ and confirmed by us, could certainly pose a barrier to liver regeneration in old animals. Indeed, recent studies have shown that shortly after PH there is a reduction in the transcriptional regulatory activity of HNF4*α* in the liver of young mice.^61, 62^ Although this decrease in HNF4*α* activity may be due to its physical redistribution on the genome, as occurs during liver development,^74^ we observed a transient but clear downregulation of HNF4*α* gene expression in the first hours post PH. This previously unrecognized response may be part of the molecular mechanisms allowing the entry of otherwise quiescent hepatocytes into the cell cycle.

The mechanisms responsible for HNF4*α* upregulation in aged mouse livers are likely to be multifarious. For instance, tissue hypoxia, a condition that develops in the liver of old animals, has been reported to stimulate HNF4*α* expression in hepatocytes.^60^ Now we found that the levels of SLU7, a gene that promotes HNF4*α* expression in the adult liver,^63^ were increased in the liver of aged mice and interestingly were downregulated upon Fibapo administration. It is worth mentioning that SLU7 expression is also rapidly reduced in mouse liver shortly after PH,^63^ with kinetics overlapping those of HNF4*α* expression reported here.

Although the identity of the endogenous signals mediating HNF4*α* downregulation after PH is currently unknown, we could induce this effect in the liver of aged mice by Fibapo treatment. Our *in vitro* experiments proved that Fibapo can directly regulate HNF4*α* gene expression, both at the transcriptional and post-transcriptional level, promoting HNF4*α* proteasomal degradation. These findings are consistent with the previously described inhibition of HNF4*α* gene expression upon pharmacological activation of the MEK-ERK pathway in liver parenchymal cells.^75^ Interestingly, we also observed that interference with autocrine FGF19/FGFR4 signaling in Hep3B cells increased HNF4*α* expression. This response supports the specificity of Fibapo effects. However, it may also have broader implications in the context of hepatocarcinogenesis, where HNF4*α* expression is consistently downregulated and FGF19 is frequently overexpressed.^5, 76^

Collectively, our observations demonstrate the efficacy of FGF19-based molecules like Fibapo as hepatoprotective and pro-regenerative agents. The two experimental models implemented in this study reproduce clinical situations for which effective treatments are lacking. The validation of these findings in models of acute liver injury and regeneration using larger animals is therefore warranted.

## Materials and methods

### Animal models

All the experimental protocols were approved and performed according to the guidelines of the Animal Care Committee of the University of Navarra. For APAP treatment mice (C57/BL/6 J, male 8–12 weeks of age) were injected intraperitoneally with 300 mg/kg or 500 mg/kg of the compound after an overnight fast. APAP (Sigma-Aldrich, St. Louis, MO, USA) was dissolved in warm PBS (55 ºC) and cooled to 37º before injection, as described.^77^ When indicated mice were killed and livers were removed to be paraffin-embedded or snap frozen in liquid N_2_. Two-thirds PH was performed in young (8–12 weeks of age) and old (12–14 months old) C57/BL/6 J male mice as previosuly described.^34^ In the APAP model, and at the indicated time points for each experiment, mice received an intravenous injection of Fibapo (2 mg/kg), synthesized upon request by GenScript (Piscataway, NJ, USA),^22^ an intraperitoneal injection of NAC (600 mg/kg) (Sigma-Aldrich), or the same volume of saline (300 *μ*l). Aged C57/BL/6 J male mice received a daily single subcutaneous injection of Fibapo (2 mg/kg) or saline for three days prior to PH. Hepatectomies were performed 24 h after the last injection as previously described.^22^ For the analysis of liver, S6 and p70S6K phosphorylation Fibapo (2 mg/kg) was injected intravenously to mice that had been fasted overnight.

### Cell culture and treatments

The human hepatocarcinoma cell line Hep3B (from ATCC) was cultured as described.^63^ Cells were treated with Fibapo in serum-free DMEM medium supplemented with 0.2% bovine serum albumin. Where indicated cells were pre-treated with the MEK1 inhibitor UO126 (10 *μ*M) (Promega, Madison, WI, USA), the proteasome inhibitor MG-132 (10 *μ*M) (Sigma-Aldrich), or the specific FGFR4 inhibitor BLU9931 (100 nM) (Cayman Chemical, Ann Arbor, MI, USA).^64^ For FGF19 knockdown Hep3B cells were transfected with FGF19-specific siRNAs (Santa Cruz Biotechnology, Santa Cruz, CA, USA), or with control siRNAs (siGL) for 48 h as previously described.^63^ Experiments were performed at least three times in duplicates. Bone marrow-derived macrophages were prepared essentially as described.^78^ To induce the proliferation of macrophages, bone marrow cells were plated in plastic plates with 10% fetal calf serum in RPMI1640 supplemented with 100 ng/ml recombinant murine macrophage-colony stimulating factor (R&D Systems, Minneapolis, MN, USA).

### Western blot analyses

Cells and liver tissues were lysed in RIPA buffer and homogenates were subjected to Western blot analysis as reported.^34, 63^ Liver tissue mitochondria were isolated as described before.^79^ Antibodies used were: anti-p-ERK1/2 (Thr202, Tyr204), anti-ERK1/2, anti-p-JNK (Thr183, Tyr185), anti-p-p70S6K (Thr389), anti-p70S6K, anti-p-S6 (Ser235, Ser236), anti-S6, anti-*β*-actin and anti-GAPDH (both used as loading controls) from Cell Signaling Technology (Beverly, MA, USA); anti-*α*-tubulin, anti-CCNE1, anti-PCNA, anti-p21 and rabbit polyclonal anti-HNF4*α* (H-171) were from Santa Cruz Biotechnology (Dallas, TX, USA); mouse monoclonal anti-HNF4*α* (PP-K9218-00) was from Perseus Proteomics Inc. (Tokyo, Japan); anti-CYP2E1 and anti-cytochrome oxidase IV were from Abcam (Cambridge, UK). Numbers shown under blot images indicate the quantification of bands intensity (averaged values) relative to controls, which were arbitrarily given the value of one.

### RNA isolation and qPCR

Total RNA from liver tissues and Hep3B cells was extracted using the automated Maxwell system from Promega. Reverse transcription was performed as described.^63^ Real-time PCRs were performed with iQ SYBR Green supermix (BioRad, Hercules, CA, USA) in a CFX96 system from BioRad as previously described.^63^ Primers are described in Supplementary Table 1.

### Hepatic triglyceride content determination

Intrahepatic TG concentration was measured by saponification in ethanolic KOH and determining triolein equivalents using a commercially available kit (BQ029A-CR, BQ Kits, San Diego, CA, USA) as described.^22^

### Histological determinations

Liver tissue samples were formalin-fixed and paraffin-embedded. Ki-67 immunostaining was performed as described.^22^ Positive Ki-67 hepatocyte number was determined in 10 liver fields per mice (× 10 amplification) using ImageJ software (NIH, Bethesda, MD, USA). To evaluate the degree of tissue necrosis H&E staining was performed and scored as previously reported.^34^ Hepatocyte cell size was determined after *β*-catenin immunostaining of paraffin sections to outline individual hepatocytes, anti-*β*-catenin antibody was from Cell Signaling Technology. To calculate hepatocyte cell size 10 fields (× 40) per tissue sample were analyzed as we previosuly described.^80^ GSH contents in liver tissues were determined as reported.^77^ For the determination of hepatic levels of BA liver tissues were homogenated in water (5 mg/500 *μ*l), sonicated and centrifuged at 10 000 × *g* for 10 min. BAs concentrations were determined in supernatants by an enzymatic/colorimetric method using a commercially available kit (Randox Laboratories, Crumlin, UK) as reported.^22^

### Serum biochemistry

Serum levels of alanine aminotransferase, aspartate aminotransferase, lactate dehydrogenase and albumin were measured as previously reported.^22, 34, 63^

### Statistical analysis

Data are means±S.E.M. Data were compared using the Student *t*-test. A *P-*value of <0.05 was considered significant. Data analyses were performed using GraphPad Prism software (GraphPad Software Inc., San Diego, USA) version 7.0 was employed for statistical analyses.

## Publisher’s Note:

Springer Nature remains neutral with regard to jurisdictional claims in published maps and institutional affiliations.

## References

[bib1] Michalopoulos GK. Advances in liver regeneration. Expert Rev Gastroenterol Hepatol 2014; 8: 897–907. 24964729 10.1586/17474124.2014.934358

[bib2] Fausto N, Campbell JS, Riehle KJ. Liver regeneration. Hepatology 2006; 43: S45–S53. 16447274 10.1002/hep.20969

[bib3] Pomfret EA, Pomposelli JJ, Gordon FD, Erbay N, Lyn Price L, Lewis WD et al. Liver regeneration and surgical outcome in donors of right-lobe liver grafts. Transplantation 2003; 76: 5–10. 12865779 10.1097/01.TP.0000079064.08263.8E

[bib4] Avila MA. Long distance calling for liver regeneration: identification of neuroendocrine signalling pathways activated after partial hepatectomy. J Hepatol 2011; 54: 403–405. 21084132 10.1016/j.jhep.2010.08.009

[bib5] Berasain C, Avila MA. Regulation of hepatocyte identity and quiescence. Cell Mol Life Sci 2015; 72: 3831–3851. 26089250 10.1007/s00018-015-1970-7 PMC11114060

[bib6] Michalopoulos GK. Hepatostat: liver regeneration and normal liver tissue maintenance. Hepatology 2016; 65: 1384–1392. 10.1002/hep.28988 27997988

[bib7] Forbes SJ, Newsome PN. Liver regeneration - mechanisms and models to clinical application. Nat Rev Gastroenterol Hepatol 2016; 13: 473–485. 27353402 10.1038/nrgastro.2016.97

[bib8] Bernal W, Lee WM, Wendon J, Larsen FS, Williams R. Acute liver failure: a curable disease by 2024? J Hepatol 2015; 62: S112–S120. 25920080 10.1016/j.jhep.2014.12.016

[bib9] Jaeschke H, Williams CD, Ramachandran A, Bajt ML. Acetaminophen hepatotoxicity and repair: the role of sterile inflammation and innate immunity. Liver Int 2012; 32: 8–20. 21745276 10.1111/j.1478-3231.2011.02501.x PMC3586825

[bib10] Du K, Ramachandran A, Jaeschke H. Oxidative stress during acetaminophen hepatotoxicity: Sources, pathophysiological role and therapeutic potential. Redox Biol 2016; 10: 148–156. 27744120 10.1016/j.redox.2016.10.001 PMC5065645

[bib11] Schmidt LE, Dalhoff K. Alpha-fetoprotein is a predictor of outcome in acetaminophen-induced liver injury. Hepatology 2005; 41: 26–31. 15690478 10.1002/hep.20511

[bib12] Apte U, Singh S, Zeng G, Cieply B, Virji MA, Wu T et al. Beta-catenin activation promotes liver regeneration after acetaminophen-induced injury. Am J Pathol 2009; 175: 1056–1065. 19679878 10.2353/ajpath.2009.080976 PMC2731124

[bib13] Rudraiah S, Manautou JE. From hepatoprotection models to new therapeutic modalities for treating liver diseases: a personal perspective. F1000Res 2016; 5(F1000 Faculty Rev): 1698. 10.12688/f1000research.8609.1 PMC4946394 27499850

[bib14] Hu B, Colletti LM. Stem cell factor and c-kit are involved in hepatic recovery after acetaminophen-induced liver injury in mice. Am J Physiol Gastrointest Liver Physiol 2008; 295: G45–G53. 18467506 10.1152/ajpgi.00024.2008 PMC2494727

[bib15] Donahower BC, McCullough SS, Hennings L, Simpson PM, Stowe CD, Saad AG et al. Human recombinant vascular endothelial growth factor reduces necrosis and enhances hepatocyte regeneration in a mouse model of acetaminophen toxicity. J Pharmacol Exp Ther 2010; 334: 33–43. 20363854 10.1124/jpet.109.163840 PMC2912052

[bib16] Ye D, Wang Y, Li H, Jia W, Man K, Lo CM et al. Fibroblast growth factor 21 protects against acetaminophen-induced hepatotoxicity by potentiating peroxisome proliferator-activated receptor coactivator protein-1α-mediated antioxidant capacity in mice. Hepatology 2014; 60: 977–989. 24590984 10.1002/hep.27060

[bib17] Stutchfield BM, Antoine DJ, Mackinnon AC, Gow DJ, Bain CC, Hawley CA et al. CSF1 restores innate immunity after liver injury in mice and serum levels indicate outcomes of patients with acute liver failure. Gastroenterology 2015; 149 1896–1909 e14. 26344055 10.1053/j.gastro.2015.08.053 PMC4672154

[bib18] Scheiermann P, Bachmann M, Goren I, Zwissler B, Pfeilschifter J, Mühl H. Application of interleukin-22 mediates protection in experimental acetaminophen-induced acute liver injury. Am J Pathol 2013; 182: 1107–1113. 23375450 10.1016/j.ajpath.2012.12.010

[bib19] Cho JY, Suh K-S, Lee HW, Cho E-H, Yang SH, Cho YB et al. Hepatic steatosis is associated with intrahepatic cholestasis and transient hyperbilirubinemia during regeneration after living donor liver transplantation. Transpl Int 2006; 19: 807–813. 16961772 10.1111/j.1432-2277.2006.00355.x

[bib20] Kele PG, van der Jagt EJ, Gouw ASH, Lisman T, Porte RJ, de Boer MT. The impact of hepatic steatosis on liver regeneration after partial hepatectomy. Liver Int 2013; 33: 469–475. 23311417 10.1111/liv.12089

[bib21] Hamano M, Ezaki H, Kiso S, Furuta K, Egawa M, Kizu T et al. Lipid overloading during liver regeneration causes delayed hepatocyte DNA replication by increasing ER stress in mice with simple hepatic steatosis. J Gastroenterol 2014; 49: 305–316. 23512345 10.1007/s00535-013-0780-7 PMC3925298

[bib22] Alvarez-Sola G, Uriarte I, Latasa MU, Fernández-Barrena MG, Urtasun R, Elizalde M et al. Fibroblast growth factor 15/19 (FGF15/19) protects from diet-induced hepatic steatosis: development of an FGF19-based chimeric molecule to promote fatty liver regeneration. Gut 2017; 66: 1818–1828. 28119353 10.1136/gutjnl-2016-312975

[bib23] Timchenko NA. Aging and liver regeneration. Trends Endocrinol Metab 2009; 20: 171–176. 19359195 10.1016/j.tem.2009.01.005

[bib24] Schmucker DL, Sanchez H. Liver regeneration and aging: a current perspective. Curr Gerontol Geriatr Res 2011; 2011: 526379. 21912543 10.1155/2011/526379 PMC3170699

[bib25] Zhu C, Ikemoto T, Utsunomiya T, Yamada S, Morine Y, Imura S et al. Senescence-related genes possibly responsible for poor liver regeneration after hepatectomy in elderly patients. J Gastroenterol Hepatol 2014; 29: 1102–1108. 24325248 10.1111/jgh.12468

[bib26] Krupczak-Hollis K, Wang X, Dennewitz MB, Costa RH. Growth hormone stimulates proliferation of old-aged regenerating liver through forkhead box m1b. Hepatology 2003; 38: 1552–1562. 14647066 10.1016/j.hep.2003.08.052

[bib27] Jin J, Wang G-L, Shi X, Darlington GJ, Timchenko NA. The age-associated decline of glycogen synthase kinase 3beta plays a critical role in the inhibition of liver regeneration. Mol Cell Biol 2009; 29: 3867–3880. 19398579 10.1128/MCB.00456-09 PMC2704742

[bib28] Jin J, Iakova P, Jiang Y, Medrano EE, Timchenko NA. The reduction of SIRT1 in livers of old mice leads to impaired body homeostasis and to inhibition of liver proliferation. Hepatology 2011; 54: 989–998. 21638299 10.1002/hep.24471 PMC3242815

[bib29] Loforese G, Malinka T, Keogh A, Baier F, Simillion C, Montani M et al. Impaired liver regeneration in aged mice can be rescued by silencing Hippo core kinases MST1 and MST2. EMBO Mol Med 2017; 9: 46–60. 27940445 10.15252/emmm.201506089 PMC5210079

[bib30] Wang X, Quail E, Hung NJ, Tan Y, Ye H, Costa RH. Increased levels of forkhead box M1B transcription factor in transgenic mouse hepatocytes prevent age-related proliferation defects in regenerating liver. Proc Natl Acad Sci USA 2001; 98: 11468–11473. 11572993 10.1073/pnas.201360898 PMC58753

[bib31] Chen W-D, Wang Y-D, Zhang L, Shiah S, Wang M, Yang F et al. Farnesoid X receptor alleviates age-related proliferation defects in regenerating mouse livers by activating forkhead box m1b transcription. Hepatology 2010; 51: 953–962. 19998409 10.1002/hep.23390 PMC3033699

[bib32] Kir S, Beddow SA, Samuel VT, Miller P, Previs SF, Suino-Powell K et al. FGF19 as a postprandial, insulin-independent activator of hepatic protein and glycogen synthesis. Science 2011; 331: 1621–1624. 21436455 10.1126/science.1198363 PMC3076083

[bib33] Potthoff MJ, Kliewer SA, Mangelsdorf DJ. Endocrine fibroblast growth factors 15/19 and 21: from feast to famine. Genes Dev 2012; 26: 312–324. 22302876 10.1101/gad.184788.111 PMC3289879

[bib34] Uriarte I, Fernández-Barrena MG, Monte MJ, Latasa MU, Chang HCY, Carotti S et al. Identification of fibroblast growth factor 15 as a novel mediator of liver regeneration and its application in the prevention of post-resection liver failure in mice. Gut 2013; 62: 899–910. 23292666 10.1136/gutjnl-2012-302945

[bib35] Kong B, Huang J, Zhu Y, Li G, Williams J, Shen S et al. Fibroblast growth factor 15 deficiency impairs liver regeneration in mice. Am J Physiol Gastrointest Liver Physiol 2014; 306: G893–G902. 24699334 10.1152/ajpgi.00337.2013 PMC4024724

[bib36] Padrissa-Altés S, Bachofner M, Bogorad RL, Pohlmeier L, Rossolini T, Böhm F et al. Control of hepatocyte proliferation and survival by Fgf receptors is essential for liver regeneration in mice. Gut 2015; 64: 1444–1453. 25416068 10.1136/gutjnl-2014-307874

[bib37] Modica S, Petruzzelli M, Bellafante E, Murzilli S, Salvatore L, Celli N et al. Selective activation of nuclear bile acid receptor FXR in the intestine protects mice against cholestasis. Gastroenterology 2012; 142 355–65 e1–e4. 22057115 10.1053/j.gastro.2011.10.028

[bib38] Luo J, Ko B, Elliott M, Zhou M, Lindhout DA, Phung V et al. A nontumorigenic variant of FGF19 treats cholestatic liver diseases. Sci Transl Med 2014; 6: 247ra100. 10.1126/scitranslmed.3009098 25080475

[bib39] Bhushan B, Walesky C, Manley M, Gallagher T, Borude P, Edwards G et al. Pro-regenerative signaling after acetaminophen-induced acute liver injury in mice identified using a novel incremental dose model. Am J Pathol 2014; 184: 3013–3025. 25193591 10.1016/j.ajpath.2014.07.019 PMC4215032

[bib40] Wan ZY, Tian JS, Tan HWS, Chow AL, Sim AYL, Ban KHK et al. Mechanistic target of rapamycin complex 1 (mTORC1) is an essential mediator of metabolic and mitogenic effects of FGF19 in hepatoma cells. Hepatology 2016; 64: 1289–1301. 27178107 10.1002/hep.28639

[bib41] Mühl H. STAT3, a key parameter of cytokine-driven tissue protection during sterile inflammation - the case of experimental acetaminophen (paracetamol)-induced liver damage. Front Immunol 2016; 7: 163. 27199988 10.3389/fimmu.2016.00163 PMC4852172

[bib42] Hanawa N, Shinohara M, Saberi B, Gaarde WA, Han D, Kaplowitz N. Role of JNK translocation to mitochondria leading to inhibition of mitochondria bioenergetics in acetaminophen-induced liver injury. J Biol Chem 2008; 283: 13565–13577. 18337250 10.1074/jbc.M708916200 PMC2376214

[bib43] Furuta K, Yoshida Y, Ogura S, Kurahashi T, Kizu T, Maeda S et al. Gab1 adaptor protein acts as a gatekeeper to balance hepatocyte death and proliferation during acetaminophen-induced liver injury in mice. Hepatology 2016; 63: 1340–1355. 26680679 10.1002/hep.28410

[bib44] Zhou M, Yang H, Learned RM, Tian H, Ling L. Non-cell-autonomous activation of IL-6/STAT3 signaling mediates FGF19-driven hepatocarcinogenesis. Nat Commun 2017; 8: 15433. 28508871 10.1038/ncomms15433 PMC5440856

[bib45] Kurosu H, Choi M, Ogawa Y, Dickson AS, Goetz R, Eliseenkova AV et al. Tissue-specific expression of betaKlotho and fibroblast growth factor (FGF) receptor isoforms determines metabolic activity of FGF19 and FGF21. J Biol Chem 2007; 282: 26687–26695. 17623664 10.1074/jbc.M704165200 PMC2496965

[bib46] Enkhbold C, Morine Y, Utsunomiya T, Imura S, Ikemoto T, Arakawa Y et al. Dysfunction of liver regeneration in aged liver after partial hepatectomy. J Gastroenterol Hepatol 2015; 30: 1217–1224. 25682855 10.1111/jgh.12930

[bib47] Sánchez-Hidalgo JM, Naranjo A, Ciria R, Ranchal I, Aguilar-Melero P, Ferrín G et al. Impact of age on liver regeneration response to injury after partial hepatectomy in a rat model. J Surg Res 2012; 175: e1–e9. 22341343 10.1016/j.jss.2011.11.1022

[bib48] Hartleb M, Barański K, Zejda J, Chudek J, Więcek A. Non-alcoholic fatty liver (NAFL) and advanced fibrosis in the elderly: results from a community-based Polish survey. Liver Int 2017. (e-pub ahead of print). 10.1111/liv.13471 28489307

[bib49] Bechmann LP, Kocabayoglu P, Sowa J-P, Sydor S, Best J, Schlattjan M et al. Free fatty acids repress small heterodimer partner (SHP) activation and adiponectin counteracts bile acid-induced liver injury in superobese patients with nonalcoholic steatohepatitis. Hepatology 2013; 57: 1394–1406. 23299969 10.1002/hep.26225

[bib50] Chiang JYL. Bile acids: regulation of synthesis. J Lipid Res 2009; 50: 1955–1966. 19346330 10.1194/jlr.R900010-JLR200 PMC2739756

[bib51] Lee YJ, Ko EH, Kim JE, Kim E, Lee H, Choi H et al. Nuclear receptor PPARγ-regulated monoacylglycerol O-acyltransferase 1 (MGAT1) expression is responsible for the lipid accumulation in diet-induced hepatic steatosis. Proc Natl Acad Sci USA 2012; 109: 13656–13661. 22869740 10.1073/pnas.1203218109 PMC3427113

[bib52] Wang M-J, Chen F, Li J-X, Liu C-C, Zhang H-B, Xia Y et al. Reversal of hepatocyte senescence after continuous *in vivo* cell proliferation. Hepatology 2014; 60: 349–361. 24711261 10.1002/hep.27094

[bib53] Wang G-L, Shi X, Salisbury E, Sun Y, Albrecht JH, Smith RG et al. Growth hormone corrects proliferation and transcription of phosphoenolpyruvate carboxykinase in livers of old mice via elimination of CCAAT/enhancer-binding protein alpha-Brm complex. J Biol Chem 2007; 282: 1468–1478. 17107955 10.1074/jbc.M608226200

[bib54] Iakova P, Awad SS, Timchenko NA. Aging reduces proliferative capacities of liver by switching pathways of C/EBPalpha growth arrest. Cell 2003; 113: 495–506. 12757710 10.1016/s0092-8674(03)00318-0

[bib55] Wang X, Kiyokawa H, Dennewitz MB, Costa RH. The Forkhead Box m1b transcription factor is essential for hepatocyte DNA replication and mitosis during mouse liver regeneration. Proc Natl Acad Sci USA 2002; 99: 16881–16886. 12482952 10.1073/pnas.252570299 PMC139238

[bib56] Walesky C, Gunewardena S, Terwilliger EF, Edwards G, Borude P, Apte U. Hepatocyte-specific deletion of hepatocyte nuclear factor-4α in adult mice results in increased hepatocyte proliferation. Am J Physiol Gastrointest Liver Physiol 2013; 304: G26–G37. 23104559 10.1152/ajpgi.00064.2012 PMC3543634

[bib57] Walesky C, Edwards G, Borude P, Gunewardena S, O'Neil M, Yoo B et al. Hepatocyte nuclear factor 4 alpha deletion promotes diethylnitrosamine-induced hepatocellular carcinoma in rodents. Hepatology 2013; 57: 2480–2490. 23315968 10.1002/hep.26251 PMC3669646

[bib58] Bonzo JA, Ferry CH, Matsubara T, Kim J-H, Gonzalez FJ. Suppression of hepatocyte proliferation by hepatocyte nuclear factor 4α in adult mice. J Biol Chem 2012; 287: 7345–7356. 22241473 10.1074/jbc.M111.334599 PMC3293558

[bib59] Walesky C, Apte U. Role of hepatocyte nuclear factor 4α (HNF4α) in cell proliferation and cancer. Gene Expr 2015; 16: 101–108. 25700366 10.3727/105221615X14181438356292 PMC5841246

[bib60] Park EY, Lee CH, Lee EK, Kim JH, Cova A, Lee SK et al. HNF4α contributes to glucose formation in aged rat hepatocytes. Exp Gerontol 2013; 48: 1518–1525. 24177414 10.1016/j.exger.2013.10.011

[bib61] Chen H, Lu S, Zhou J, Bai Z, Fu H, Xu X et al. An integrated approach for the identification of USF1-centered transcriptional regulatory networks during liver regeneration. Biochim Biophys Acta 2014; 1839: 415–423. 24686121 10.1016/j.bbagrm.2014.03.010

[bib62] Jiao H, Zhu Y, Lu S, Zheng Y, Chen H. An integrated approach for the identification of HNF4α-centered transcriptional regulatory networks during early liver regeneration. Cell Physiol Biochem 2015; 36: 2317–2326. 26279436 10.1159/000430195

[bib63] Elizalde M, Urtasun R, Azkona M, Latasa MU, Goñi S, Garcia-Irigoyen O et al. Splicing regulator SLU7 is essential for maintaining liver homeostasis. J Clin Invest 2014; 124: 2909–2920. 24865429 10.1172/JCI74382 PMC4071377

[bib64] Hagel M, Miduturu C, Sheets M, Rubin N, Weng W, Stransky N et al. First selective small molecule inhibitor of FGFR4 for the treatment of hepatocellular carcinomas with an activated FGFR4 signaling pathway. Cancer Discov 2015; 5: 424–437. 25776529 10.1158/2159-8290.CD-14-1029

[bib65] Bhushan B, Borude P, Edwards G, Walesky C, Cleveland J, Li F et al. Role of bile acids in liver injury and regeneration following acetaminophen overdose. Am J Pathol 2013; 183: 1518–1526. 24007882 10.1016/j.ajpath.2013.07.012 PMC3814573

[bib66] Espeillac C, Mitchell C, Celton-Morizur S, Chauvin C, Koka V, Gillet C et al. S6 kinase 1 is required for rapamycin-sensitive liver proliferation after mouse hepatectomy. J Clin Invest 2011; 121: 2821–2832. 21633171 10.1172/JCI44203 PMC3223822

[bib67] Berasain C, García-Trevijano ER, Castillo J, Erroba E, Lee DC, Prieto J et al. Amphiregulin: an early trigger of liver regeneration in mice. Gastroenterology 2005; 128: 424–432. 15685553 10.1053/j.gastro.2004.11.006

[bib68] Wang X, Bhattacharyya D, Dennewitz MB, Kalinichenko VV, Zhou Y, Lepe R et al. Rapid hepatocyte nuclear translocation of the Forkhead Box M1B (FoxM1B) transcription factor caused a transient increase in size of regenerating transgenic hepatocytes. Gene Expr 2003; 11: 149–162. 14686788 10.3727/000000003108749044 PMC5991162

[bib69] Paranjpe S, Bowen WC, Mars WM, Orr A, Haynes MM, DeFrances MC et al. Combined systemic elimination of MET and epidermal growth factor receptor signaling completely abolishes liver regeneration and leads to liver decompensation. Hepatology 2016; 64: 1711–1724. 27397846 10.1002/hep.28721 PMC5074871

[bib70] Bhushan B, Chavan H, Borude P, Xie Y, Du K, McGill MR et al. Dual role of epidermal growth factor receptor in liver injury and regeneration after acetaminophen overdose in mice. Toxicol Sci 2017; 155: 363–378. 28123000 10.1093/toxsci/kfw213 PMC6020708

[bib71] James LP, Lamps LW, McCullough S, Hinson JA. Interleukin 6 and hepatocyte regeneration in acetaminophen toxicity in the mouse. Biochem Biophys Res Commun 2003; 309: 857–863. 13679052 10.1016/j.bbrc.2003.08.085

[bib72] Sanchis-Gomar F, Pareja-Galeano H, Santos-Lozano A, Garatachea N, Fiuza-Luces C, Venturini L et al. A preliminary candidate approach identifies the combination of chemerin, fetuin-A, and fibroblast growth factors 19 and 21 as a potential biomarker panel of successful aging. Age (Dordr) 2015; 37: 9776. 25911468 10.1007/s11357-015-9776-y PMC4409588

[bib73] Wang X, Krupczak-Hollis K, Tan Y, Dennewitz MB, Adami GR, Costa RH. Increased hepatic Forkhead Box M1B (FoxM1B) levels in old-aged mice stimulated liver regeneration through diminished p27Kip1 protein levels and increased Cdc25B expression. J Biol Chem 2002; 277: 44310–44316. 12221098 10.1074/jbc.M207510200

[bib74] Alder O, Cullum R, Lee S, Kan AC, Wei W, Yi Y et al. Hippo signaling influences HNF4A and FOXA2 enhancer switching during hepatocyte differentiation. Cell Rep 2014; 9: 261–271. 25263553 10.1016/j.celrep.2014.08.046 PMC4612615

[bib75] Hatzis P, Kyrmizi I, Talianidis I. Mitogen-activated protein kinase-mediated disruption of enhancer-promoter communication inhibits hepatocyte nuclear factor 4alpha expression. Mol Cell Biol 2006; 26: 7017–7029. 16980607 10.1128/MCB.00297-06 PMC1592892

[bib76] Alvarez-Sola G, Uriarte I, Latasa MU, Urtasun R, Barcena-Varela M, Elizalde M et al. Fibroblast growth factor 15/19 in hepatocarcinogenesis. Dig Dis 2017; 35: 158–165. 28249259 10.1159/000450905

[bib77] Baulies A, Ribas V, Núñez S, Torres S, Alarcón-Vila C, Martínez L et al. Lysosomal cholesterol accumulation sensitizes to acetaminophen hepatotoxicity by impairing mitophagy. Sci Rep 2015; 5: 18017. 26657973 10.1038/srep18017 PMC4676017

[bib78] Inaba K, Inaba M, Romani N, Aya H, Deguchi M, Ikehara S et al. Generation of large numbers of dendritic cells from mouse bone marrow cultures supplemented with granulocyte/macrophage colony-stimulating factor. J Exp Med 1992; 176: 1693–1702. 1460426 10.1084/jem.176.6.1693 PMC2119469

[bib79] Rogers GW, Brand MD, Petrosyan S, Ashok D, Elorza AA, Ferrick DA et al. High throughput microplate respiratory measurements using minimal quantities of isolated mitochondria. PLoS ONE 2011; 6: e21746. 21799747 10.1371/journal.pone.0021746 PMC3143121

[bib80] Argemí J, Kress TR, Chang HCY, Ferrero R, Bértolo C, Moreno H et al. X-box binding protein 1 regulates unfolded protein, acute-phase, and DNA damage responses during regeneration of mouse liver. Gastroenterology 2017; 152 1203–1216 e15. 28082079 10.1053/j.gastro.2016.12.040

