# Supplementary figures and images for: Engineered fibroblast growth factor 19 protects from acetaminophen-induced liver injury and stimulates aged liver regeneration in mice
Source: Cell Death Dis. 2017 Oct 5;8(10):e3083–. doi: 10.1038/cddis.2017.480 (PMC5682649; doi:10.1038/cddis.2017.480)

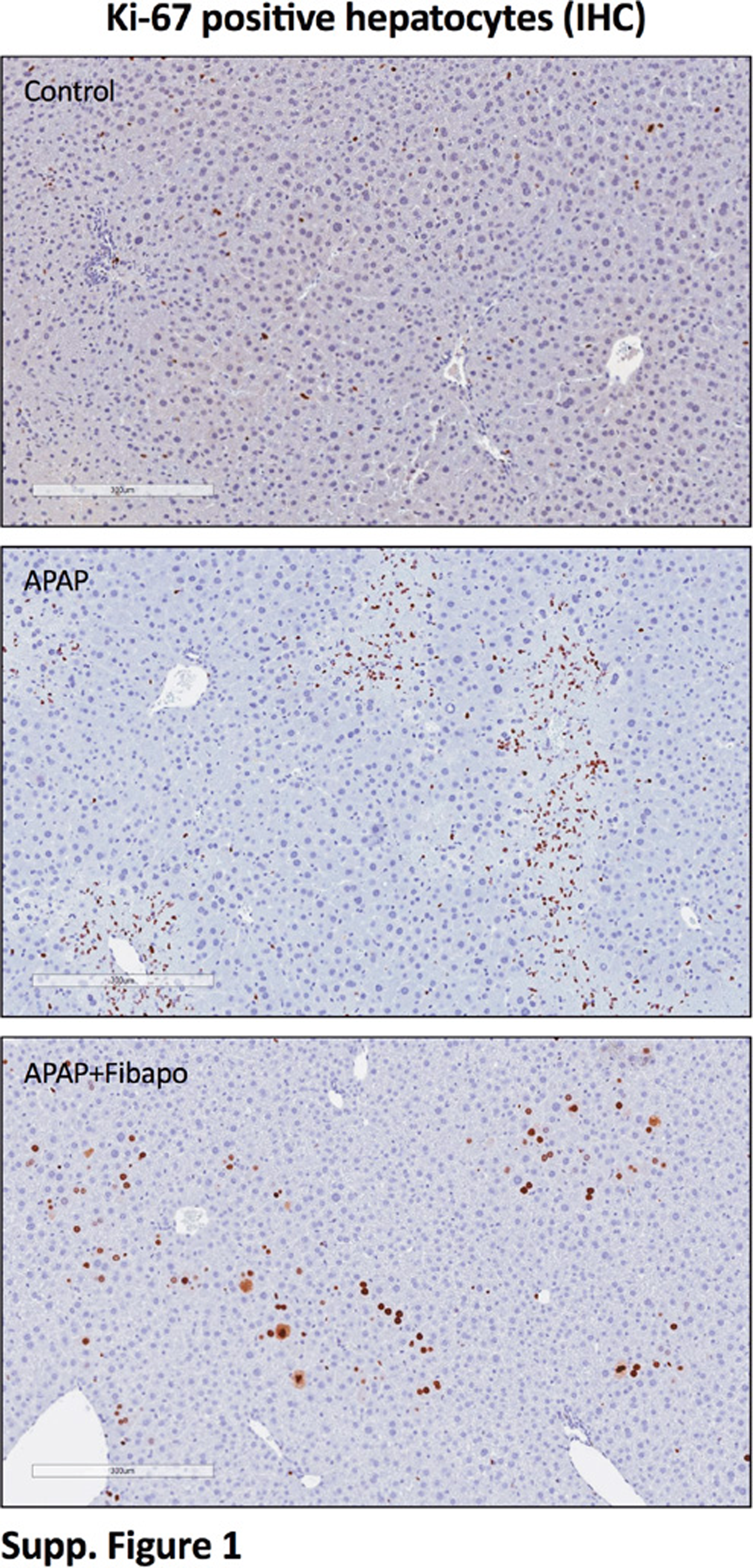

Supplement: Supplementary Figure 1 [file cddis2017480x1.tif]

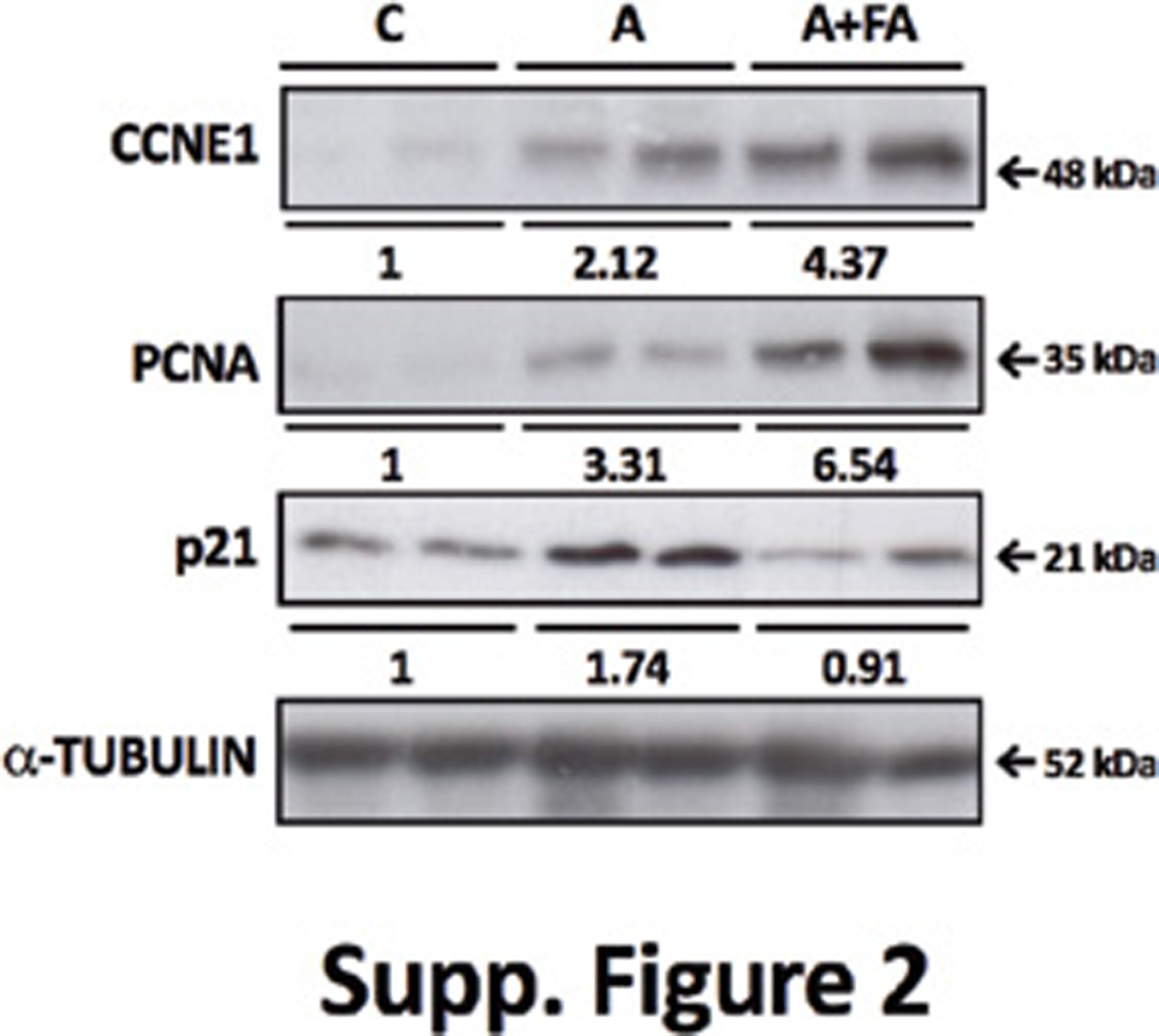

Supplement: Supplementary Figure 2 [file cddis2017480x2.tif]

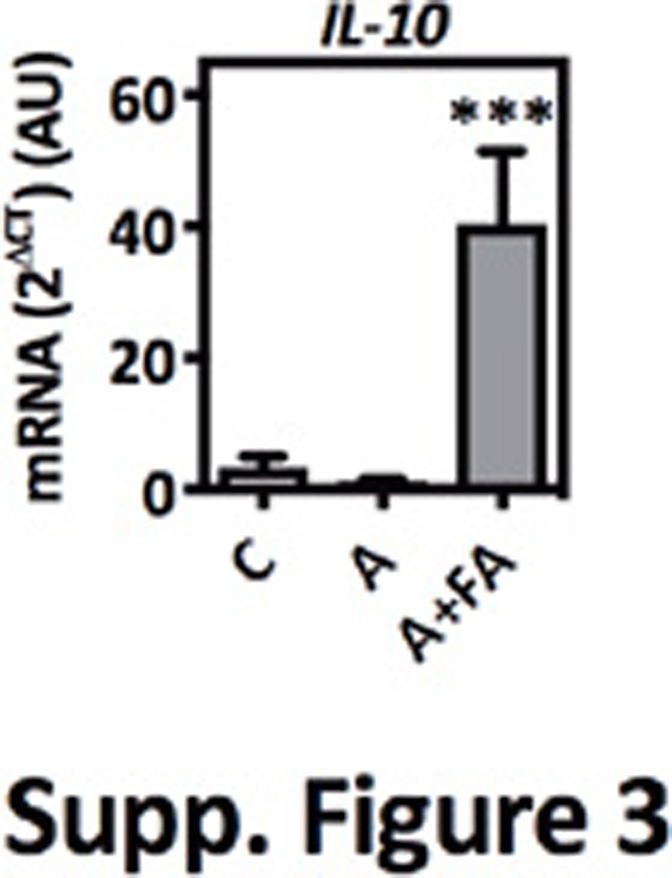

Supplement: Supplementary Figure 3 [file cddis2017480x3.tif]

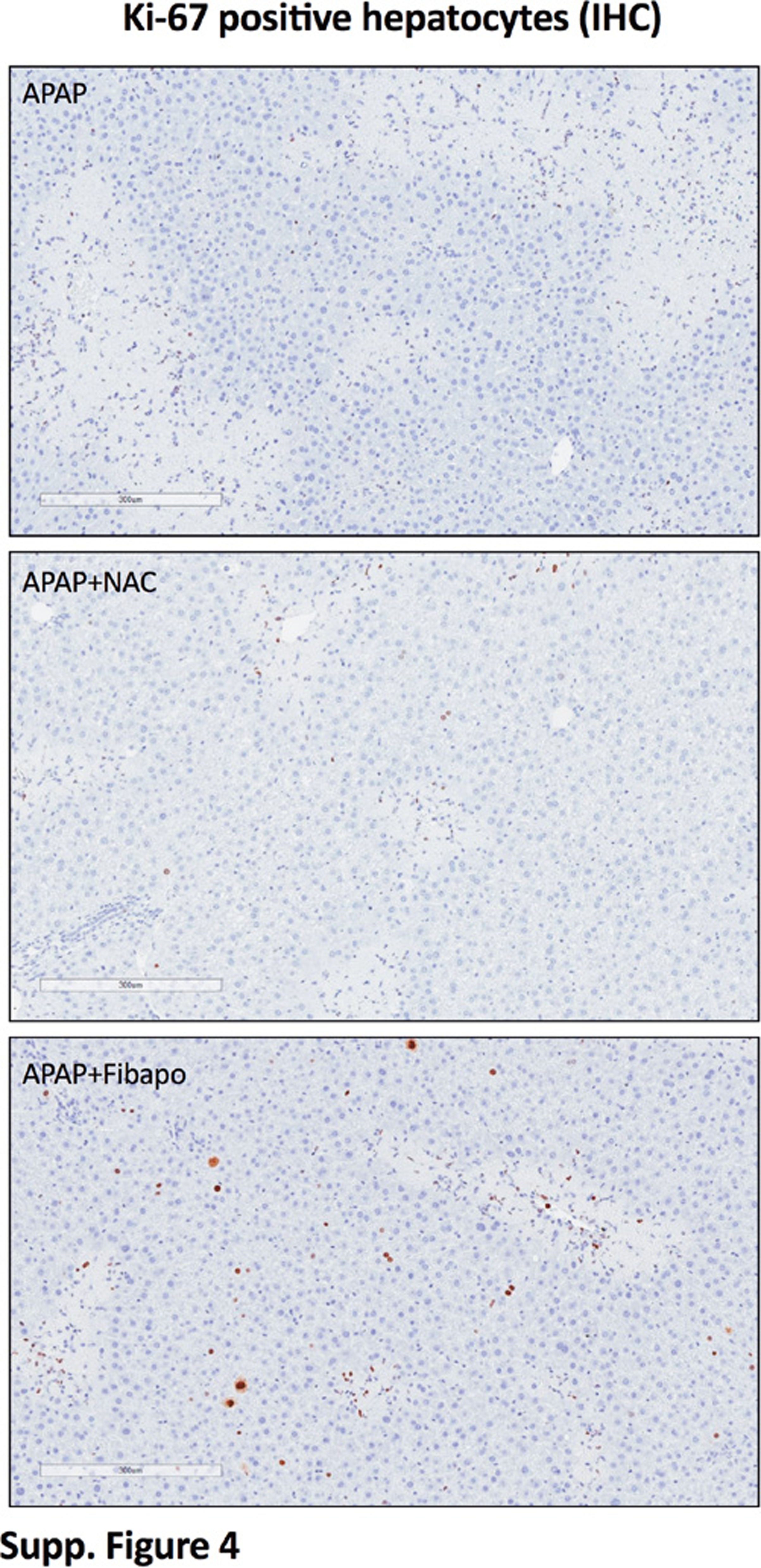

Supplement: Supplementary Figure 4 [file cddis2017480x4.tif]

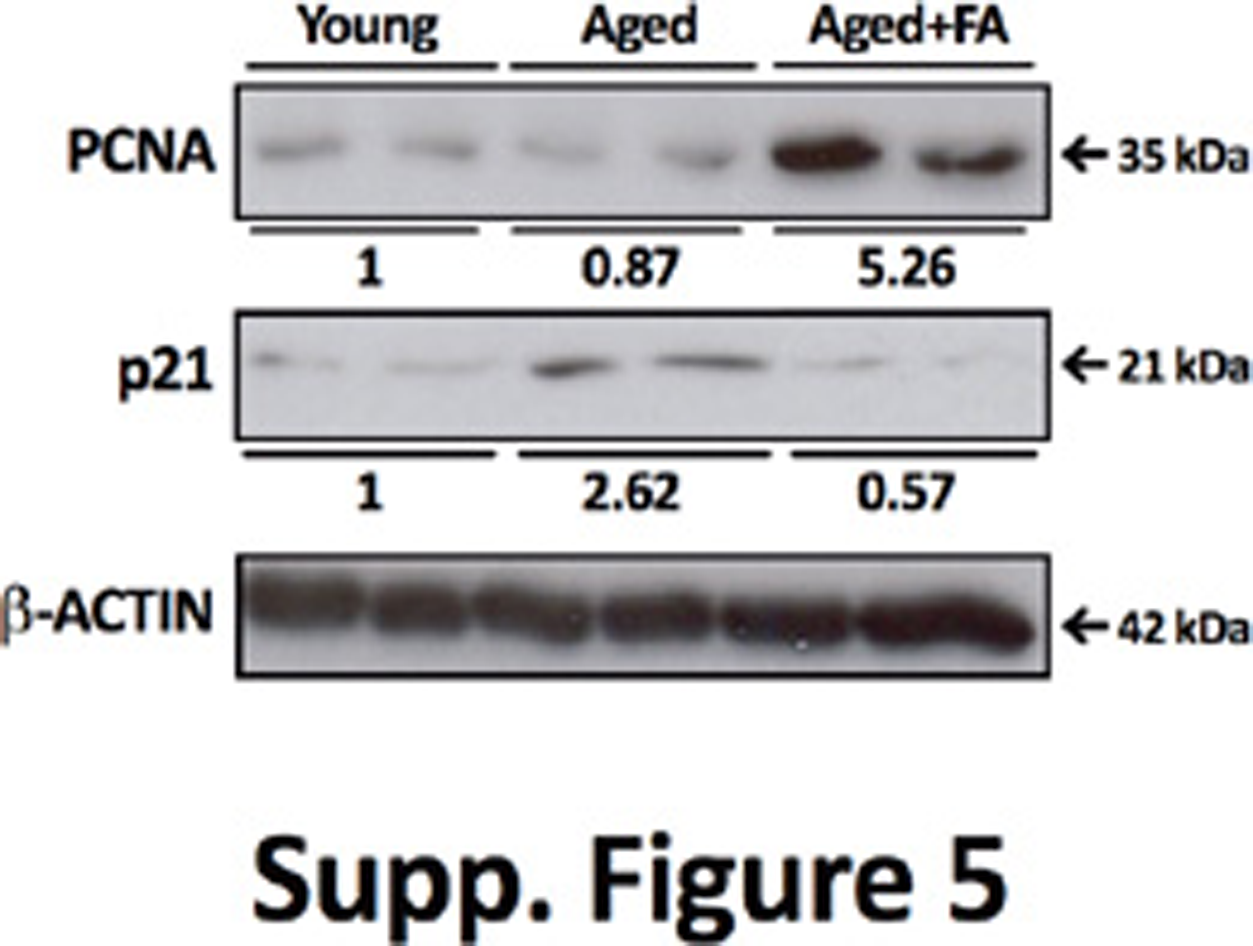

Supplement: Supplementary Figure 5 [file cddis2017480x5.tif]

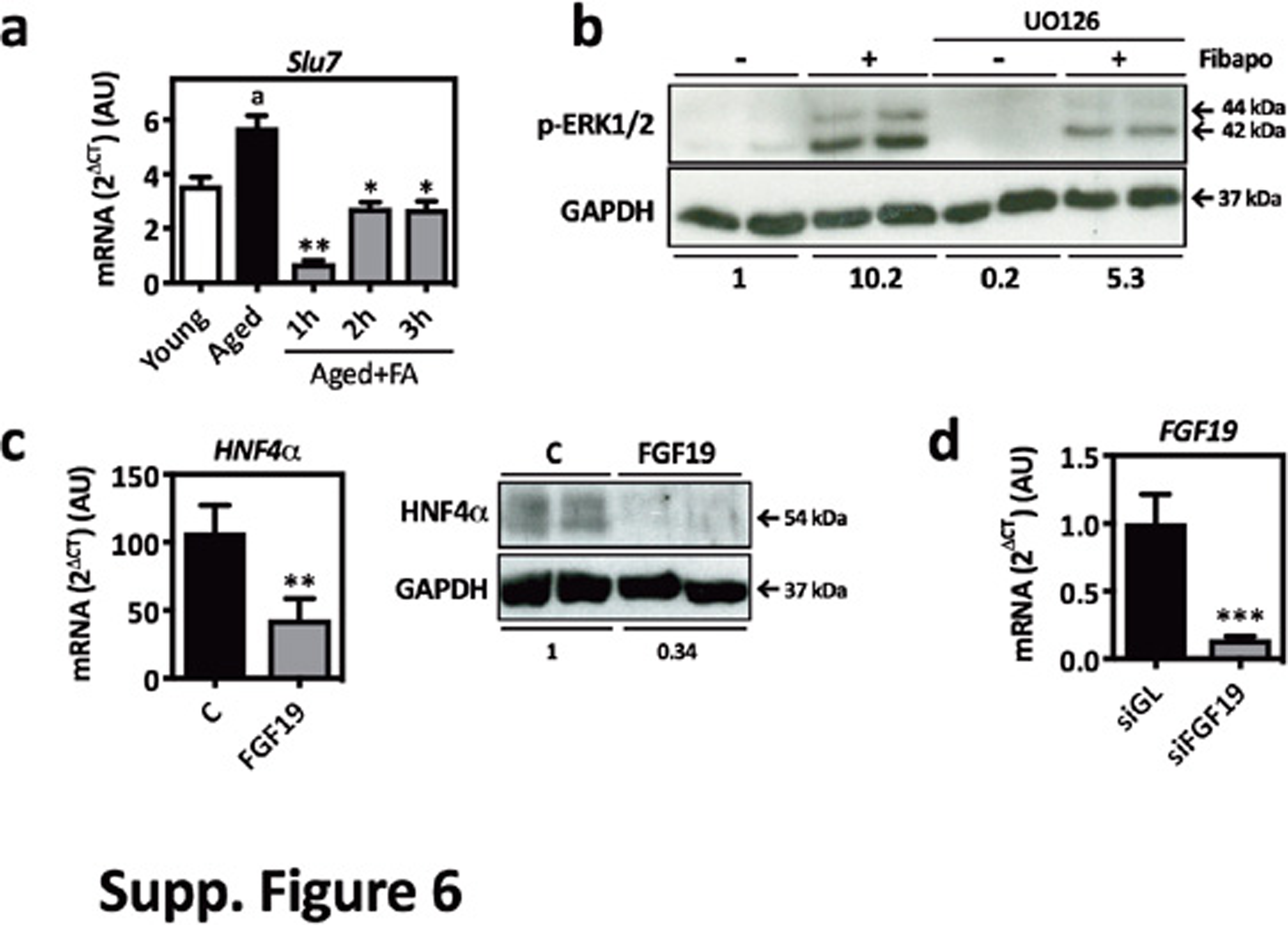

Supplement: Supplementary Figure 6 [file cddis2017480x6.tif]
