# Supplementary material for: Engineered fibroblast growth factor 19 protects from acetaminophen-induced liver injury and stimulates aged liver regeneration in mice
Source: Cell Death Dis. 2017 Oct 5;8(10):e3083–. doi: 10.1038/cddis.2017.480 (PMC5682649; doi:10.1038/cddis.2017.480)
Supplement: Supplementary Figure Legends [file cddis2017480x7.docx]

**Supplementary Figure 1.** Ki-67 immunohistochemical analysis in liver tissue sections from control mice, mice treated with APAP (300 mg/kg) or mice that 2, 10 and 24 h after APAP injection received three doses of Fibapo (APAP+Fibapo). Mice were sacrificed 36 h after APAP administration. Notice that most Ki-67 positive cells in liver tissue samples from APAP treated mice are inflammatory cells that infiltrate necrotic areas. Representative images are shown.

**Supplementary Figure 2.** Western blot analysis of Ccne1, PCNA and p21 protein levels in liver tissue samples from mice treated as described in Figure 1. Blots were probed with anti-α-tubulin antibodies to show equal loading of gels. Representative blots are shown.

**Supplementary Figure 3.**  Quantitative PCR analysis of *IL-10* mRNA levels in the livers of control mice (C) and mice treated with APAP (A) or with APAP plus Fibapo (A+FA) as described for Figure 1. ****P*<0.001 *vs* control mice.

**Supplementary Figure 4.** Ki-67 immunohistochemical analysis in liver tissue sections from mice treated with APAP (500 mg/kg), mice that 6 and 24 h after APAP injection received two doses of NAC (APAP+NAC) or two doses of Fibapo (APAP+Fibapo). Liver samples were obtained from surviving mice 48 h after APAP administration. Representative images are shown.

**Supplementary Figure 5.** Western blot analysis of PCNA and p21 protein levels in liver tissue samples from mice treated as described in Figure 5. Representative blots are shown.

**Supplementary Figure 6.** (**a**) Quantitative PCR analysis of *SLU7* mRNA levels in the liver of young mice, control aged mice, and aged mice at the indicated time-point after Fibapo administration. a *P*<0.05 *vs* young mice, **P*<0.05 and ***P*<0.01 *vs* aged mice. (**b**) Effect of Fibapo on p-ERK1/2 levels in Hep3B cells in the presence of the MEK-ERK signaling inhibitor UO126 analyzed by western blotting. Cells were pretreated with UO126 (10 μM) for 45 min prior to Fibapo (50 ng/ml) addition and were lysed after 8 h of treatment. Representative blots are shown. (**c**) Left panel shows *HNF4α* mRNA levels analyzed by quantitative PCR in Hep3B cells treated for 12 h with FGF19 (50 ng/ml). Right panel shows HNF4α protein levels determined by western blotting in Hep3B cells treated with 50ng/ml of FGF19 for 12 h. A representative blot is shown. (**d**) FGF19 mRNA levels determined by quantitative PCR in Hep3B cells transfected with an FGF19 specific siRNA (siFGF19) or a control siRNA (siGL) 72 h after transfections. ****P*<0.001 *vs* control.
