# Supplementary material for: Engineered fibroblast growth factor 19 protects from acetaminophen-induced liver injury and stimulates aged liver regeneration in mice
Source: Cell Death Dis. 2017 Oct 5;8(10):e3083–. doi: 10.1038/cddis.2017.480 (PMC5682649; doi:10.1038/cddis.2017.480)
Supplement: Supplementary Table 1 [file cddis2017480x8.docx]

| **Gene** |  | **Sequence** |
| --- | --- | --- |
| *Bcl-xL* | Forward | 5´-GCGCGGGAGGTGATTCCCATGGC-3´ |
|  | Reverse | 5´-CATGCCCGTCAGGAACCAGCG-3´ |
| *β-Klotho* | Forward | 5´-TTGCTGCTTCATCTCCACTC-3´ |
|  | Reverse | 5´-TGGTTACAGCTGTCTCTCAC-3´ |
| *Ccnb2* | Forward | 5´-CCATTCATGTGGATGAAGCAG-3´ |
|  | Reverse | 5´-GTCCATTTATATCTCTTCCATCTAAG-3´ |
| *Ccne1* | Forward | 5´-TGATGAAGGCCCTTAAGTGG-3´ |
|  | Reverse | 5´-GGCCACTTGGACATAGACAT-3´ |
| *Cdc25b* | Forward | 5´-CCCTTCCCTGTTTTCCTTTC-3´ |
|  | Reverse | 5´-ACACACACTCCTGCCATAGG-3´ |
| *c-met* | Forward | 5´-CGATCAGCAGTCTGTGCATT-3´ |
|  | Reverse | 5´-ACAGCCGGAAGAGTTTCTCA-3´ |
| *Cyp7a1* | Forward | 5’-GCTGTGGTAGTGAGCTGTTGCA-3’ |
|  | Reverse | 5’-CACAGCCCAGGTATGGAATCA-3’ |
| *Dhfr* | Forward | 5´-CCTGGTTCTCCATTCCTGAG-3´ |
|  | Reverse | 5´-GCCTGGGTATTCTGGGAGA-3´ |
| *Fgfr1c* | Forward | 5´-GCCAGACAACTTGCCGTATG-3´ |
|  | Reverse | 5´-ATTTCCTTGTCGGTGGTATT-3´ |
| *Fgfr4* | Forward | 5´-GCCTCCGACAAGGATTTGGCA-3´ |
|  | Reverse | 5´-GAGTGCAGACACCCAGCAGGT-3´ |
| *Foxm1b* | Forward | 5´-CACTTGGATTGAGGACCACTT-3´ |
|  | Reverse | 5´-GTCGTTTCTGCTGTGATTCC-3´ |
| *H3F3A* | Forward | 5’-AAAGCCGCTCGCAAGAGTGCG-3’ |
|  | Reverse | 5’-ACTTGCCTCCTGCAAAGCAC-3’ |
| *Hgf* | Forward | 5´-ATCATTGGTAAAGGAGGCAGC-3´ |
|  | Reverse | 5´-AATTCCAAGGCTGGCATTTG-3´ |
| *HNF4α p1* | Forward | 5´-GCGTGGAGGCAGGGAGAATGC-3´ |
|  | Reverse | 5´-GCCCTTGCAGCCGTCACAGC-3´ |
| *Hnf4α p1* | Forward | 5´-GCGTGGGTAGGGGAGAATGC-3´ |
|  | Reverse | 5´-CCGGTCGCCACAGATGGCGC-3´ |
| *Il-6* | Forward | 5´-CAGTTGCCTTCTTGGGACTG-3´ |
|  | Reverse | 5´-GTTCATACAATCAGAATTGC-3´ |
| *p16* | Forward | 5´-GGGGTTTCGCCCAACGCCCCGA-3´ |
|  | Reverse | 5´-TGCAGCACCACCAGCGTGTCC-3´ |
| *p21* | Forward | 5´-CGGTGGAACTTTGACTTCGT-3´ |
|  | Reverse | 5´-TCTGCGCTTGGAGTGATAGA-3´ |
| *Il-10* | Forward | 5´-CTGCACCCACTTCCCAG-3´ |
|  | Reverse | 5´-AGAAATCGATGACAGCG-3´ |
| *Il-22* | Forward | 5´-GTCAACCGCACCTTTATGCT-3´ |
|  | Reverse | 5´-GTCAGGAAAGGCACCACCT-3´ |
| *Pparγ2* | Forward | 5’-GCTGTTATGGGTGAAACTCTG-3’ |
|  | Reverse | 5’-GAATAATAAGGTGGAGATGCAGG-3’ |
